# Relationship between the Pathogenic Representatives of Periodontal Pockets Microbiocenosis in Patients with Periodontitis with Varying Degrees of Severity 

**Published:** 2011

**Authors:** O.A. Zorina, A.A. Kulakov, O.A. Boriskina, D.V. Rebrikov

**Affiliations:** Central Research Institute of Dentistry and Oral Surgery; Vavilov Institute of General Genetics, Russian Academy of Sciences; Scientific Production Enterprise «DNA Technology», JSC

**Keywords:** ecosystem, habitat, periodontitis, periodontopathogenic microflora of periodontal pockets, quantitative polymerase chain reaction

## Abstract

Periodontitis is a common disease that is considered to be a manifestation of the distortion of the ratio between the normal and conditionally pathogenic microflora of periodontal pockets. In this study, the ratio between the six most important periodontal pathogens and the total microflora of the periodontal pocket in healthy individuals and patients with varying severity of periodontitis was ascertained by quantitative real-time PCR. It was ascertained that the relative content of*Porphyromonas gingivalis*,*Prevotella intermedia*, and*Tannerella forsythensis*(*Bacteroides forsythus*) persistently develops in the total microflora of the periodontal pocket upon progressing periodontitis; this value is higher than that in the control group by more than two orders of magnitude upon a severe degree of chronic generalized periodontitis.

## INTRODUCTION 

In terms of the composition of microorganisms, the oral cavity is one of the most sophisticated ecosystems of the human organism. Saliva, gingival fluid, periodontal pocket, biofilm, and a number of other habitats contain approximately 700 different types of microorganisms [1, 2], which can be divided conditionally into three large groups: 1) normoflora, 2) conditionally pathogenic, and 3) pathogenic microorganisms [3].

A number of exogenous and endogenous factors condition the bacterial profile of biocenosis of the oral cavity. The protective mechanisms of the host organism have a considerable effect on the virulence of the conditionally pathogenic and pathogenic microorganisms in each habitat. 

It is common knowledge that the distortion of the ratio between the normal and conditionally pathogenic flora leads to the development of dysbacterioses and is characterized by a relative decrease in the content of lacto- and bifidobacteria. The common disease of periodontitis is one of the manifestations of this disbalance. The essential role in the development of periodontitis is known to belong to *Porphyromonas gingivalis* , *Treponema denticola* , *Tannerella forsythensis* ( *Bacteroides forsythus* ), *Fusobacterium spp.* , and a number of other microorganisms [4–6].

Advances in molecular biology techniques, in particular the methods of qualitative and quantitative assessment of the nucleic acid content, have made it possible to determine both the composition and the relative amount of microorganisms in various sub-habitats of the oral cavity rapidly and with a high degree of accuracy [6–8]. A distinctive feature of modern molecular and genetic methods (in particular, quantitative PCR) is their high sensitivity and the possibility for a quantitative determination of anaerobic microorganisms (which is often non-feasible when using conventional culturing methods) [9]. Real-time PCR allows for the simultaneous determination of the qualitative and quantitative composition of microbiota in any habitat of the oral cavity selected.

This work is aimed at a quantitative assessment of the ratio between the most significant (according to [9–11]) representatives of the periodontopathogenic microflora of periodontal pockets: *Aggregatibacter actinomycetemcomitans* , *Porphyromonas gingivalis* , *Prevotella intermedia* , *Tannerella forsythensis* ( *Bacteroides forsythus* ), *Treponema denticola* , *Candida albicans* in healthy people and in patients with periodontitis with varying degrees of severity.

## EXPERIMENTAL 

**Table 1:** The age structure of the examined groups (%)

Age	Healthy controls (*n* = 30)	Mild degree (*n* = 10)	Average degree (*n* = 29)	Severe degree (*n* = 35)
Under 35 years old	21	80	24	17
35–44 years old	27	20	35	29
44–55 years old	33	0	31	34
Over 55 years old	19	0	10	20

**Table 2:** The proportion of positive samples depending on the group of healthy individuals or patients with CGP (%)

Microorganism name	Healthy controls (*n* = 30)	Mild degree (*n* = 10)	Average degree (*n* = 29)	Severe degree (*n* = 35)
*A. actinomycetemcomitans*	10	33	48	43
*P. gingivalis*	47	70	66	86
*P. intermedia*	33	70	59	71
*T. forsythensis*	53	80	97	100
*T. denticola*	60	80	83	83
*C. albicans*	10	40	38	60

The study was carried out in the Periodonthology Department of the Central Research Institute of Dentistry and Oral Surgery (Russia). A total of 104 individuals aged 18–65 without severe somatic pathology were examined. 

The absence of a dentogingival junction served as the major criterion for the diagnosis of chronic generalized periodontitis (CGP). The degree of severity was determined based on the depth of periodontal pockets and the degree of destruction of bone tissue. Thus, the depth of periodontal pockets was less than 3 mm in cases with a mild degree of CGP; the X-ray pattern confirming signs of initial destruction of the inter-dental septa. 

The depth of periodontal pockets varied from 3 to 6 mm in patients with a moderate degree of CGP; I–II degree of pathologic tooth mobility was frequently revealed during the examination. According to the data obtained by X-ray examination, the destruction of the cortical plate and bone tissue of interdental septa was equal to 1/2–1/3 of the length of the tooth root. 

Severe CGP was characterized by the presence of periodontal pockets more than 6 mm deep, II–III degree pathological tooth mobility, destruction of the cortical plate and bone tissue by more than 1/3 of the length of the tooth root was revealed by X-ray examination. 

The control group consisted of 30 individuals (12 males and 18 females) aged 28–55, without complaints and visible pathological changes in periodontal tissues. The data on the age structure of the groups are listed in *Table 1* .

Microflora of the intact periodontium and periodontal pockets was examined using sterile paper endodontic pins (size no. 25), which were submerged in a gingival sulcus or a pathological pocket until it reached bottom. It was subsequently kept there for 10 s. The pins were then placed into a test tube with a physiological solution, cooled, and transferred to the laboratory. A duplicate sample was taken from each patient. 

In order to reveal infectious agents and determine the genomic DNA of the patient (as a normalization index), DNA was extracted from the biological material using the “Proba-GS” kits (DNA Technology JSC, Russia) according to the enclosed manual. The method is based on sorbing DNA on a carrier, washing out the impurities, and eluting nucleic acids from the sorbent. Due to cell lysing by strong chaotropic agents, “Proba GS” destroys the cells with different types of cell walls (gram-positive/gram-negative bacteria, fungi) with almost equal efficacy. The “Proba GS” kit can be used to extract the genomic DNA of eukaryotes as well (it was used as the normalization index). 

The previously designed reagent kits, consisting of specific primers and a specific fluorescence-labeled destructible sample (TaqMan type) to six periodontopathogenic agents ( *Aggregatibacter actinomycetemcomitans* , *Porhpyromonas gingivalis* , *Prevotella intermedia* , *Tannerella forsythensis* ( *Bacteroides forsythus* ), *Treponema denticola* , and *Candida albicans* ) were used in this work. PCR with detection of the conservative region of the 16S rRNA gene was performed simultaneously with the determination of pathogenic microorganisms to estimate the total bacterial mass in the sample. The copy number of the genome equivalents of each type of bacteria (and the total bacterial mass) was normalized per amount of human genomic DNA (a fragment of the growth hormone receptor gene) in order to compare the abundance of pathogens in the samples.

**Fig. 1:**
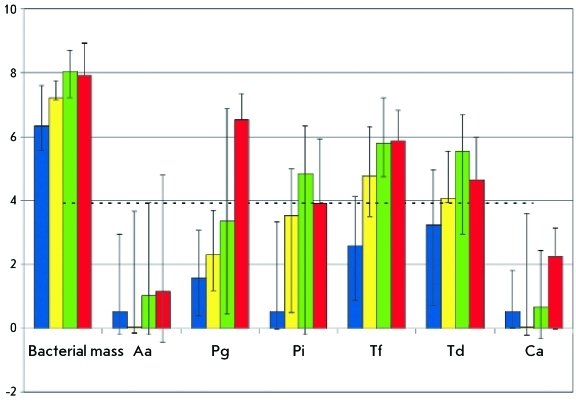
The relative number of microorganisms in healthy individuals (blue columns) and in patients with CGP of varying degrees of severity (yellow columns – mild, green – medium, and red – severe and aggressive periodontitis). The median values with 25–75 percentile error bars are shown. The data are normalized with respect to the content of human genomic DNA (dotted line). The logarithm of the content of this component in the sample (in arbitrary units) is plotted along the *Y* axis. Bacterial mass is the total number of bacteria, Aa – *A. actinomycetemcomitans* , Pg – *P. gingivalis* , Pi – *P. intermedia* , Tf – *T. forsythensis* , Td – *T. denticola* , and CA – *C. albicans* .

PCR was performed on a DTprime detecting amplifier (OOO DNA Technology, Russia). The results of the reaction were taken into consideration using the software of the DTprime amplifier. The normalized values corresponding to the level of abundance of each microorganism were calculated using the ∆∆Ct method. 

## RESULTS AND DISCUSSION 

The sample to be analyzed was divided into four groups depending on disease severity: 1) healthy control ( *n* = 30); 2) mild degree of CGP ( *n* = 10); 3) moderate degree of CGP ( *n* = 29); and 4) severe degree of CGP ( *n* = 35); where n is the number of individuals in a group.

The results of revealing pathogens in each group are listed in Table 2. It is evident that with the progression of the disease, there exists a tendency towards an increase in the number of positive samples with respect to all microorganisms. The published data on the frequency of detecting periodontopathogens in healthy individuals and in patients with CGP differ considerably [10, 12–14]; however, in most cases a similar tendency is reported. Hence, Braga *et al* . [12] detected *P. intermedia* in 80.0% of healthy individuals ( *n* = 30) and in 90.0% of patients with CGP ( *n* = 30); *P. gingivalis* was detected in 46.6% of healthy individuals and in 70.0% of patients with CGP; and *A. actinomycetemcomitans* was detected in all individuals in both groups. The differences in the frequencies of detected pathogens can be accounted for by the features of group formation, possible (in certain cases, planned) strain specificity, and the varying sensitivities of the test systems used. It should also be noted that the detection of a pathogenic microorganism in each of the individuals in the control group may attest to contamination of the laboratory with products of earlier reactions; a consideration that should alert the authors.

We used the new approach to quantitatively assess the composition of dental cavity microbiocenoses; therefore, we did not manage to find much published data on the relative content of the pathogens studied. No reliable differences were revealed by Hyvärinen *et al* . [15], who determined the relative content of *A. actinomycetemcomitans* , *P. gingivalis* , *P. intermedia* , *T. forsythensis* and *T. denticola* in saliva samples from patients with periodontitis and healthy individuals.

The results of determining the relative amount of microorganisms in healthy individuals and in patients with varying degrees of severity of CGP are presented in *Figure.* In order to reduce the amount of biomaterial used for the study to a common denominator, the data were normalized with respect to the content of the unique fragment of human genomic DNA (the fragment of the growth hormone receptor gene) [16, 17]. It is evident from *Figure* that as the disease progresses, there exists a tendency towards an increase in the content of bacteria in general (the “bacterial mass” index) and the content of most representatives of pathogenic microflora (the relative content of pathogens increasing at an anticipatory rate). *P. gingivalis* , *P. intermedia* , and *T. forsythensis* are the leaders in growth with progressing periodontitis; their content in the total bacterial mass persistently increasing by a factor of more than 100.

The obtained data attest to the possibility of using a quantitative assessment of the ratio between the pathogenic representatives of microbiocenosis of periodontal pockets as a diagnostic tool to predict the development of periodontitis. The determination of the ratio between *P. gingivalis* , *P. intermedia* , *T. forsythensis* , the total bacterial mass, and the genomic DNA of the patient can be recommended as a variant of diagnosticum.
